# Myocarditis as an immune-related adverse event following treatment with ipilimumab and nivolumab combination therapy for metastatic renal cell carcinoma: a case report

**DOI:** 10.1186/s13256-021-03097-6

**Published:** 2021-10-15

**Authors:** Yasuyuki Miyauchi, Hirohito Naito, Hiroyuki Tsunemori, Ryosuke Tani, Yusuke Hasui, Yuichi Miyake, Tetsuo Minamino, Ryo Ishikawa, Yoshio Kushida, Reiji Haba, Mikio Sugimoto

**Affiliations:** 1 Department of Urology, Faculty of Medicine, Kagawa University, 1750-1 Ikenobe, Miki-cho. Kita-gun, Kagawa, 761-0793 Japan; 2 Department of CardioRenal and CerebroVascular Medicine, Faculty of Medicine, Kagawa University, Kagawa, Japan; 3 Department of Diagnostic pathology, Kagawa University Hospital, Kagawa, Japan

**Keywords:** Immunotherapy, Immune checkpoint inhibitors, Nivolumab, Ipilimumab, Immune-related adverse events, Myocarditis, Renal cell carcinoma

## Abstract

**Background:**

Immune checkpoint inhibitors are new immunotherapy drugs globally used for many malignancies, including renal cell carcinoma. Myocarditis as an immune-related adverse event is rare but highly fatal, suggesting that its frequency may be higher than reported. This paper describes a case of myocarditis that developed asymptomatically following ipilimumab and nivolumab combination therapy for renal cell carcinoma.

**Case presentation:**

A 71-year-old Asian man who presented to hospital with fever, fatigue, and weight loss of approximately 10 kg within 2 months was diagnosed with Xp.11.2 translocation renal cell carcinoma. Computed tomography revealed multiple lung masses, mediastinal lymph node enlargement, and a level II tumor thrombus reaching the inferior vena cava (cT3bN0M1; International Metastatic Renal Cell Carcinoma Database Consortium, poor risk). Ipilimumab/nivolumab combination therapy was started as induction therapy. The patient experienced acute interstitial nephritis as an immune-related adverse event after treatment initiation; however, a good response to steroid therapy was observed. The antitumor effect of the immunotherapy was notable. Although he experienced pulmonary embolism, it seemed asymptomatic and harmless; thus, a second infusion was introduced. From the eighth day, he demonstrated rapidly worsening cardiogenic shock with asymptomatic electrocardiographic changes and drastic drop in cardiac biomarkers, and a diagnosis of myocarditis as an immune-related adverse event was made. Although immediate methylprednisolone mini-pulse therapy followed by tapered prednisolone prevented mortality, extensive myocardial fibrosis with marked ejection fraction decline persisted as a sequela. Consequently, follow-up without treatment was instituted; however, much of the tumor response initially observed was maintained over several months.

**Conclusion:**

Physicians treating patients with immune checkpoint inhibitors should be aware of their potentially life-threatening cardiotoxic effects. This study emphasized the importance of a high index of suspicion, prompt diagnosis, and early intervention in patients who present with cardiac abnormalities and possible myocarditis after receiving immunotherapy.

## Background

The combination of the anti-CTLA-4 monoclonal antibody, “ipilimumab,” and anti-PD-1 monoclonal antibody, “nivolumab,” is a revolutionary therapeutic approach and improves outcomes in patients with advanced-stage renal cell carcinoma [1]. However, this approach is associated with a wide spectrum of side effects known as immune-related adverse events (irAEs), which involve the damage of nontarget organs and are thought to arise from the aberrant activation of autoreactive T cells [2]. In recent years, with the increased use of these agents for a wide range of malignancies, myocarditis has been reported as a rare but important irAE and is associated with high mortality if not detected and treated early [3]. Herein, we present a case of advanced renal cell carcinoma in which drug-induced myocarditis rapidly worsened after immunotherapy. The myocarditis was asymptomatic and was successfully treated.

## Case presentation

**Fig. 1:**
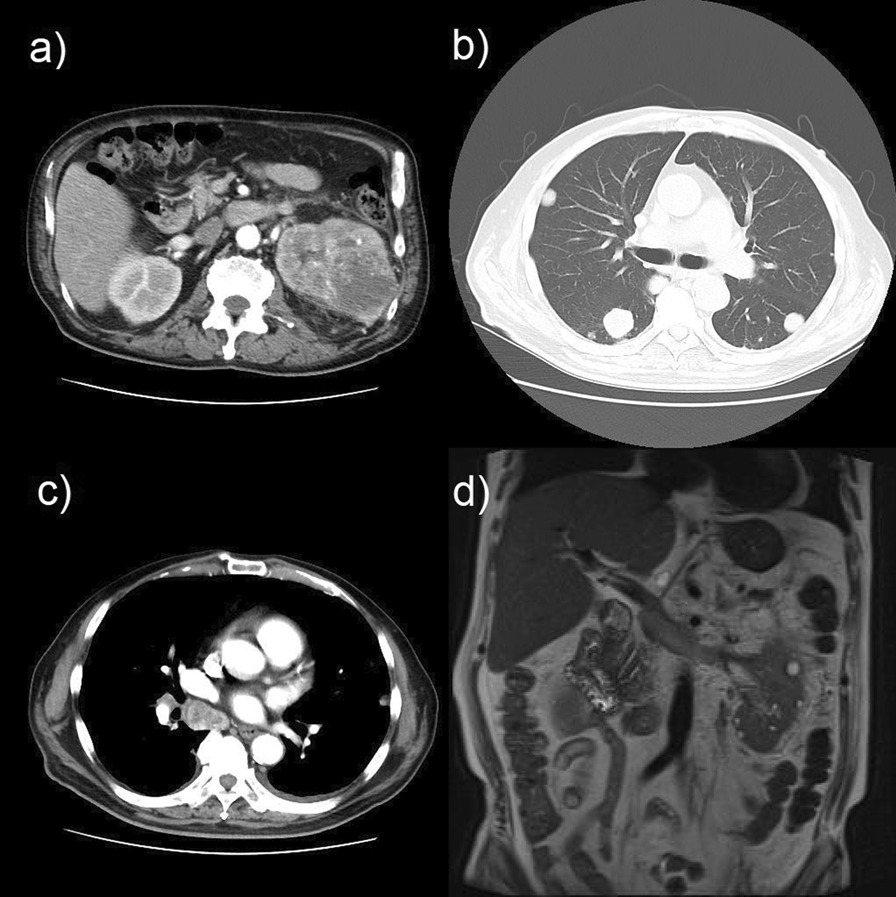
Primary and metastatic lesion suggestive of renal cell carcinoma at the first visit. Computed tomography shows a left renal mass (**a**), multiple lung masses (**b**), and mediastinal lymph node enlargement (**c**). Magnetic resonance imaging shows a level II tumor thrombus reaching the inferior vena cava (**d**)

A 71-year-old Asian man with a history of hypertension, diabetes mellitus type 2, and hyperuricemia was admitted to the hospital with fever, fatigue, and weight loss of approximately 10 kg in 2 months. The patient’s family history was unremarkable, and he had a history of smoking for about 40 years and did not have a drinking habit. Computed tomography (CT) showed a left renal mass, multiple lung masses, and mediastinal lymph node enlargement. Magnetic resonance imaging (MRI) showed a level II tumor thrombus reaching the inferior vena cava. The patient was referred to our department for multidisciplinary treatment (Fig. 1). Renal tumor biopsy revealed a diagnosis of Xp.11.2 translocation renal cell carcinoma (cT3bN0M1; International Metastatic Renal Cell Carcinoma Database Consortium; poor risk), and the patient received ipilimumab (1 mg/kg) and nivolumab (240 mg) as initial therapy for metastatic renal cell carcinoma.

**Fig. 2:**
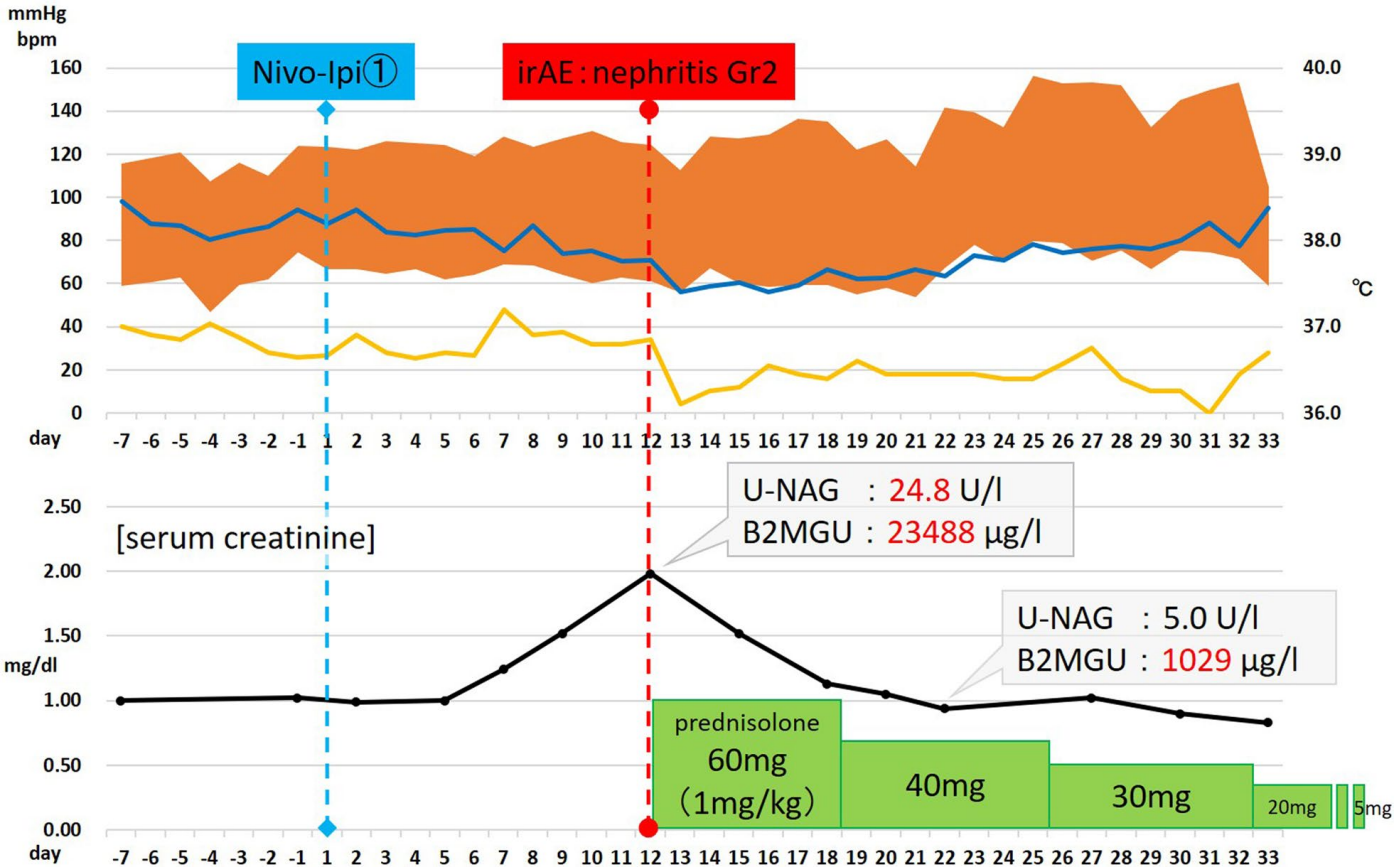
Details of the first cycle of ipilimumab/nivolumab combination therapy including kidney injury as immune-related adverse events management

Although renal function was normal at the start of the immunotherapy, the serum creatinine level was gradually elevated to 1.98 mg/dL from the seventh day of the first infusion. The patient was not taking nephrotoxic drugs and was otherwise asymptomatic. A non-contrast CT scan confirmed the absence of any obstacle in the urinary tract, while marked regression was observed in all lesions. Urine biochemistry examination showed acute tubular disorder (*N*-acetyl glucosaminidase, 24.8 U/L; β2-microglobulin, 23,488 µg/L) and a negative immunological examination led to the clinical suspicion of acute interstitial nephritis as an irAE. This renal injury was treated with intravenous prednisolone at a daily dose of 1 mg/kg, followed by oral prednisolone. Serum creatinine level and urinary biochemistry findings were also rapidly normalized with good response to steroid therapy (Fig. 2). The dose of corticosteroids was progressively tapered to 5 mg daily as maintenance. After 50 days of the first cycle, CT revealed that the primary tumor and lung metastasis showed partial response, while the mediastinal lymph node and inferior vena cava tumor thrombus demonstrated complete response. However, pulmonary embolism was noted simultaneously, and the patient was admitted and started on anticoagulation therapy with heparin. Because the respiratory and circulatory dynamics were stable and considering the achieved cancer control, a second cycle of ipilimumab/nivolumab combination therapy was started.

**Fig. 3:**
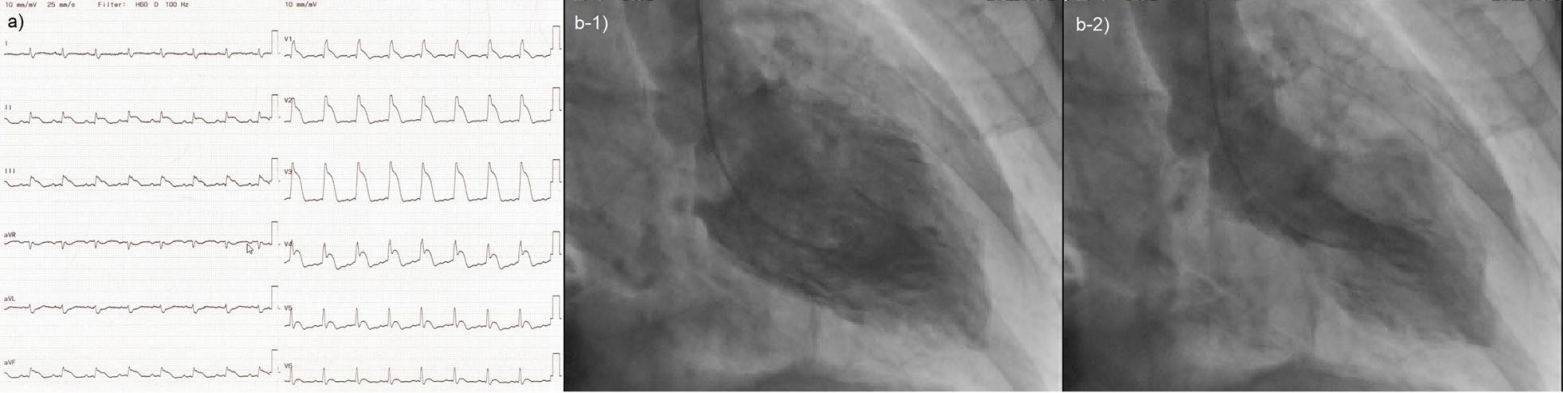
Electrocardiography findings (**a**) and ventriculogram during diastole (**b-1**) and systole (**b-2**) from immune checkpoint inhibitors-associated myocarditis. Twelve-lead electrocardiogram showing segment elevation in all leads (**a**). While the left ventricular apex in these two images appears nearly akinetic, the remaining left ventricle seems hyperkinetic (**b**)

**Fig. 4:**
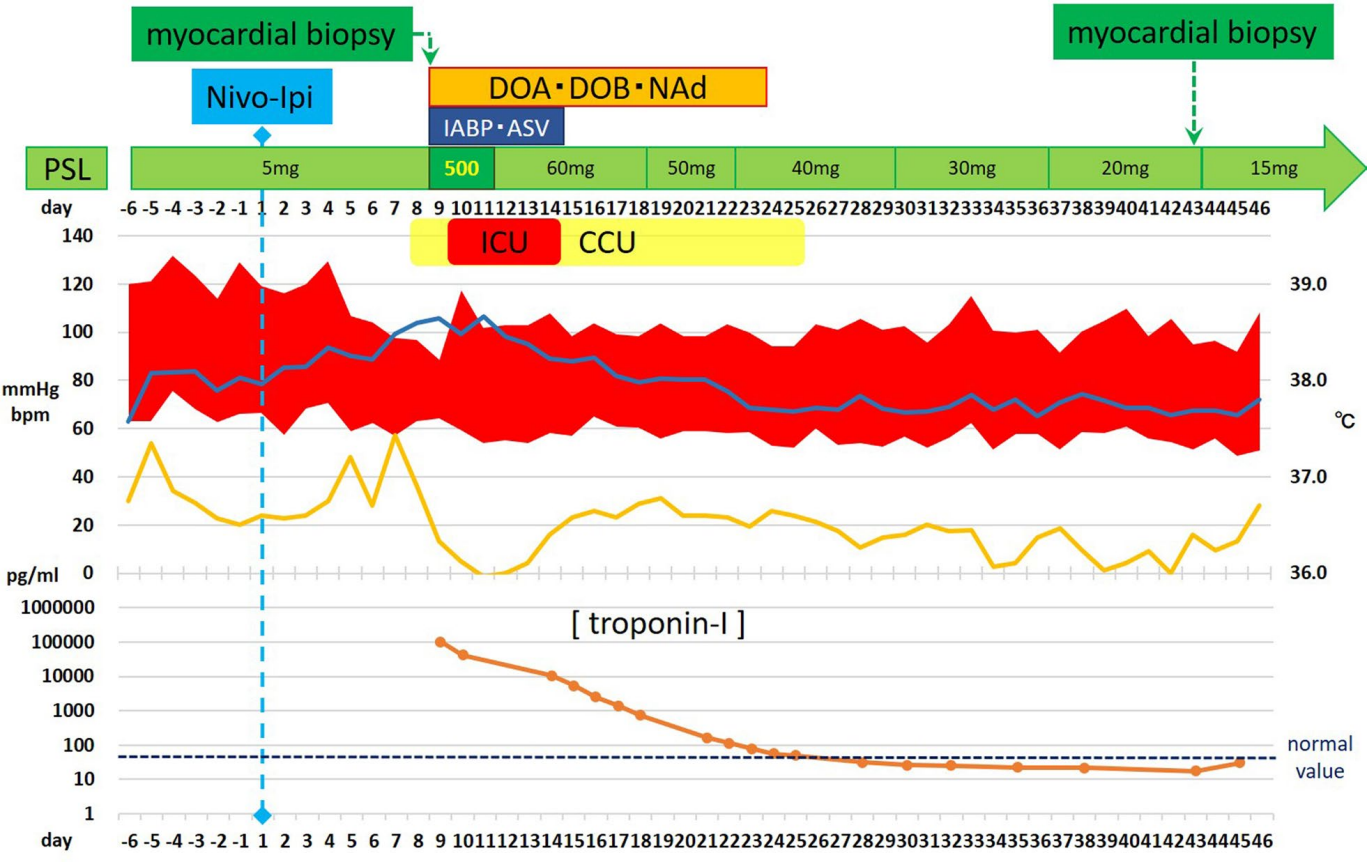
Details of the second cycle of ipilimumab/nivolumab combination therapy including myocarditis as immune-related adverse events management

**Fig. 5:**
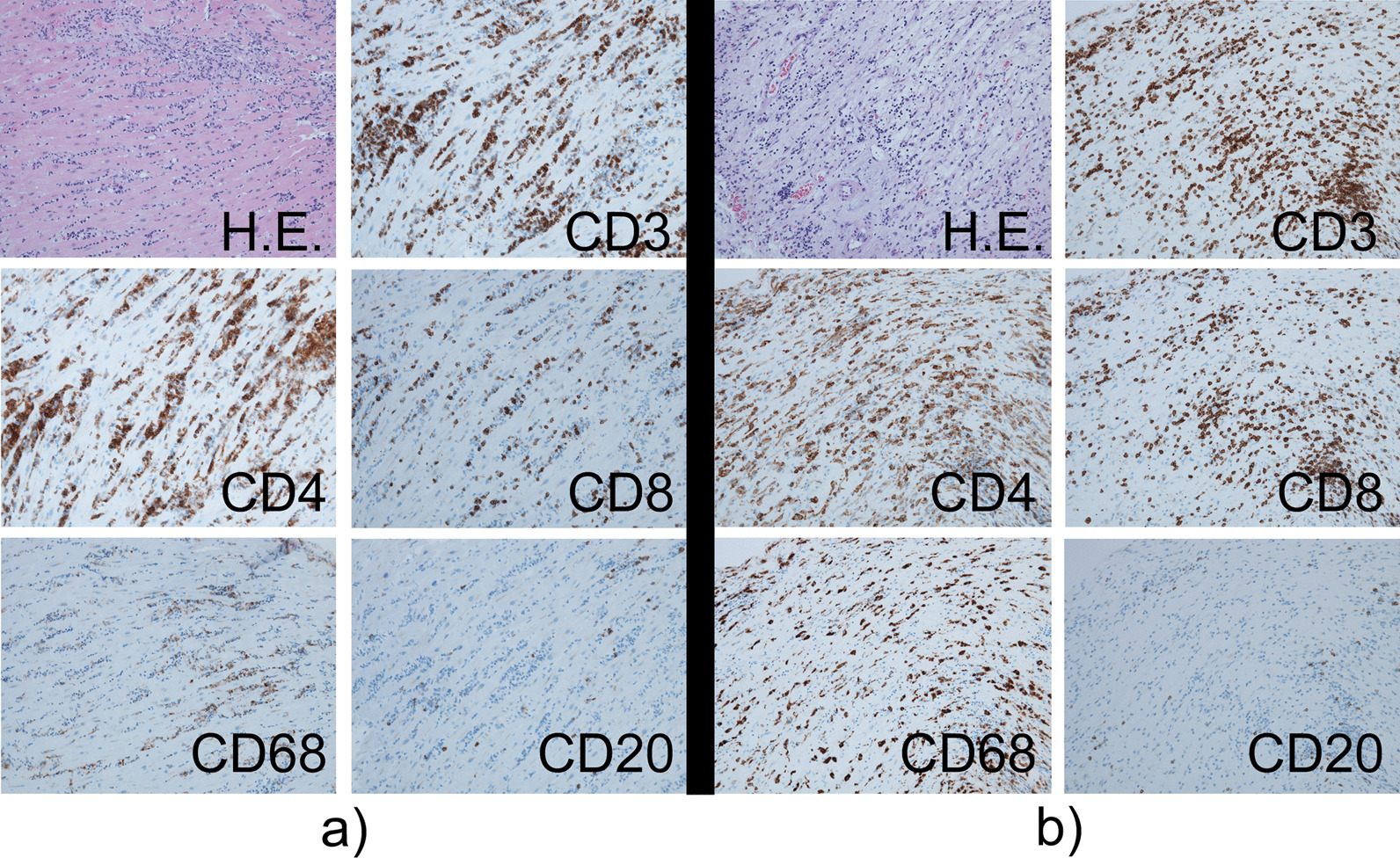
Photomicrographs of the endomyocardial biopsy sample before and after treatment. Hematoxylin–eosin staining and immunohistochemical staining of sections of the interventricular septum demonstrating staining with anti-CD3, anti-CD4, anti-CD8, anti-CD68, and anti-CD20 antibodies. All images are displayed at 20× the original magnification. Histologic findings show lymphocytic infiltration in the myocardium comprising CD3 positive T lymphocytes, many of which were positive for CD4 compared with CD8, abundance of CD68-positive macrophage infiltrate, and only rare B lymphocytes before treatment (**a**). Despite intense treatment, the remaining prominent inflammatory infiltrate consisted of CD3+, CD4+, and CD8+ T cells, and CD68+ macrophages, suggesting smoldering myocarditis (**b**)

**Fig. 6:**
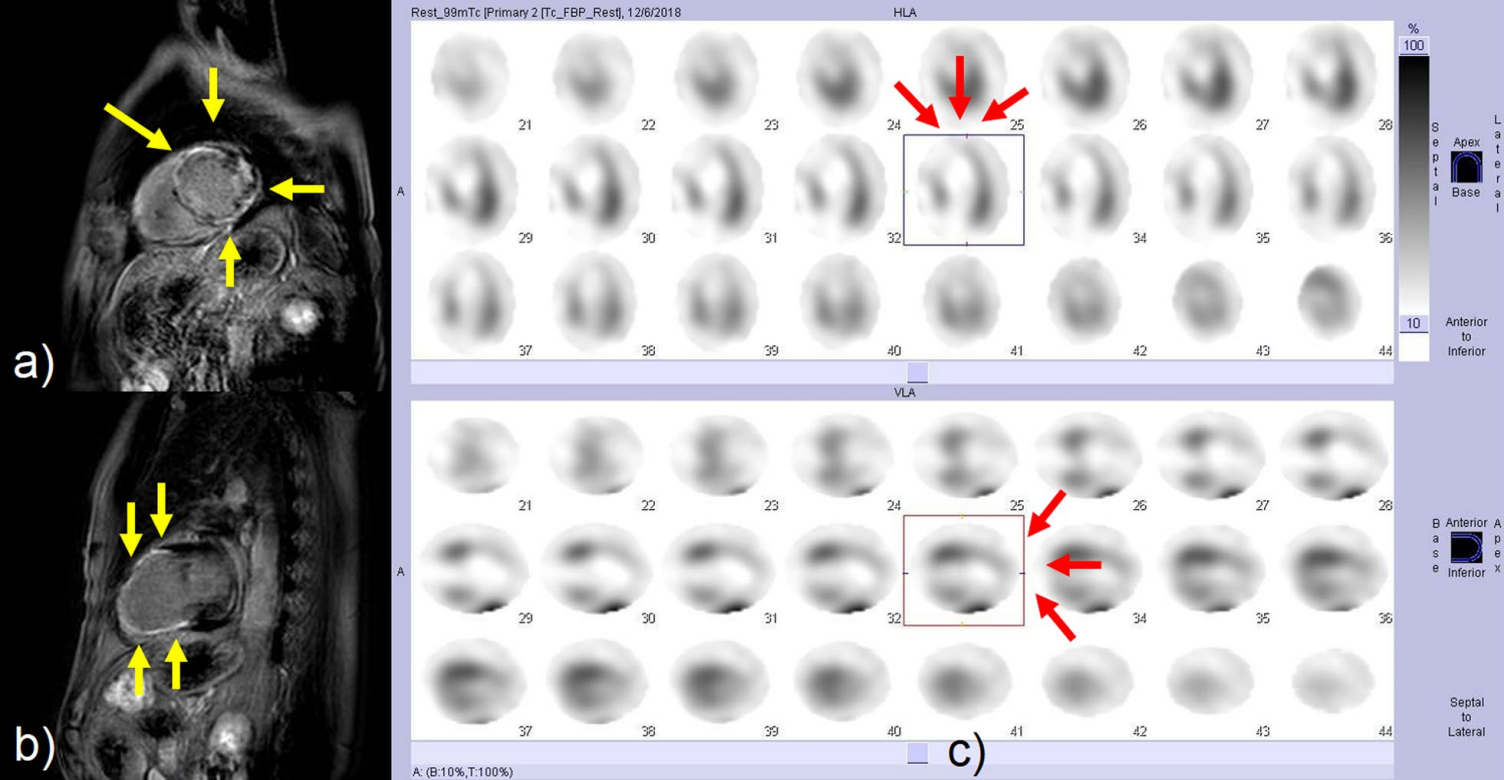
Cardiac magnetic resonance imaging (MRI) and myocardial scintigraphy in the chronic phase. Late gadolinium enhancement in the short axis (**a**) and two-chamber (**b**) views show diffuse fibrosis of the left ventricular wall especially in the inferior and anterior wall (yellow arrows). Decreased viability may be commonly observed on the anterior wall and septum of the left ventricular myocardium from the middle to the apex (red arrows) (**c**)

**Fig. 7:**
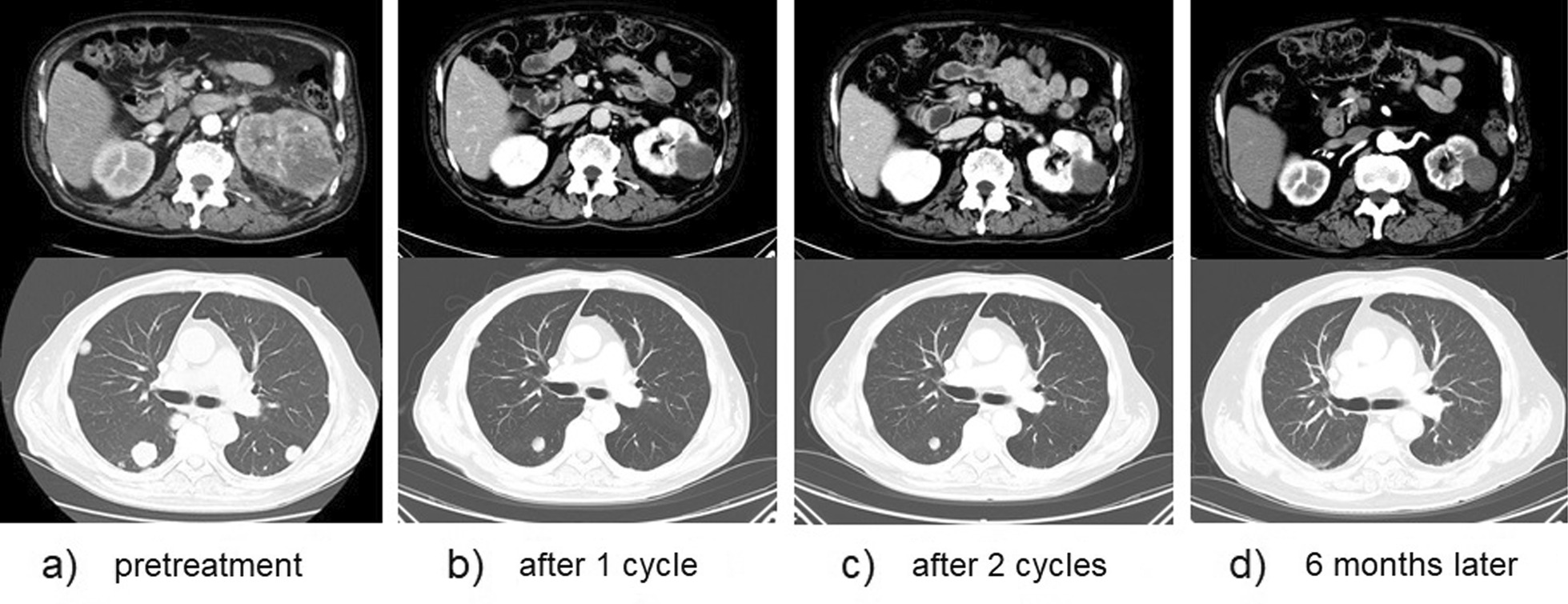
Therapeutic effect on the primary lesion and lung metastasis under immunotherapy

**Fig. 8:**
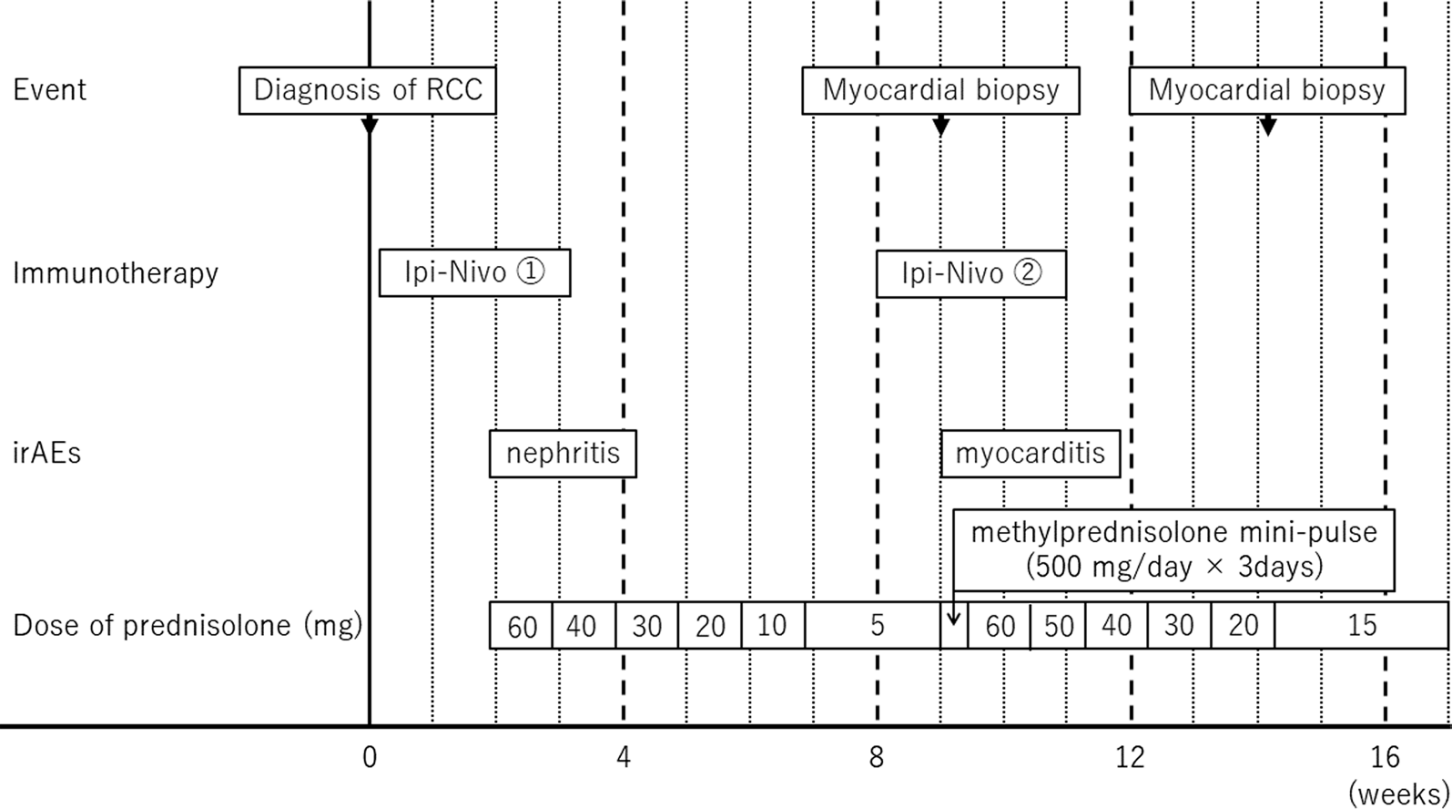
Summary of the clinical course of the present case. *RCC* renal cell carcinoma, *Ipi* ipilimumab, *Nivo* nivolumab, *irAEs* immune-related adverse events

Four days after the second infusion, the patient experienced an asymptomatic but sustainable mild rise in serum creatine phosphokinase. From the evening of the seventh day to the morning of the eighth day, ST-segment elevation was observed in the cardiac monitor despite the absence of any complaint. Hence 12-lead electrocardiography (ECG) was performed, and ST-segment elevation was seen in all leads (Fig. 3a). Laboratory analysis revealed a high level of inflammatory reaction and elevation of myocardial biomarkers (creatine kinase MB-isozyme, 23, 488 U/L; troponin-I, 100,680 pg/mL; NT-proBNP, 15,964 pg/mL), and the patient was immediately referred to cardiovascular internal medicine. Echocardiography showed an akinetic apex, a hypokinetic septum, and an ejection fraction of 50% (baseline 2 months previously was 72%). Emergency cardiac catheterization excluded relevant coronary disease, and left ventriculography showed that the left ventricular apex was nearly akinetic and the remaining left ventricle was hyperkinetic (Fig. 3b). The differential diagnoses at this point included Takotsubo cardiomyopathy triggered by viral infection and myocarditis due to autoimmunological causes. To monitor for hemodynamic failure, the patient was admitted to the coronary care unit. Twelve hours after admission to the care unit, he complained of chest tightness and shortness of breath. His hemodynamics rapidly worsened due to cardiogenic shock, and echocardiography revealed widespread cardiac akinesis except for the back wall. To maintain and improve cardiorespiratory function, dopamine, dobutamine, and noradrenaline were infused, an intra-aortic balloon pump was inserted, and adaptive servo ventilation therapy was initiated. The clinical oncology unit was consulted, and they noted that the clinical presentation was suggestive of myocarditis as an irAE. Momentarily, right heart catheterization was performed and myocardial biopsy was also performed for histological analysis. Thereafter, a 3-day methylprednisolone mini-pulse (500 mg/day) therapy was administered followed by prednisolone administration at 1 mg/kg. Nevertheless, the patient’s condition worsened, and he was admitted to the intensive care unit for percutaneous cardiopulmonary support. Although he experienced notable arrhythmia such as ventricular fibrillation, his hemodynamics gradually improved with troponin I decrease, and he returned to the general ward after treatment in the high care unit for 18 days (Fig. 4). Serological analysis of paired serum samples for cardiotropic viruses was negative. The myocardial biopsy sample taken during the acute phase demonstrated marked lymphocytic infiltration with a predominance of CD4-positive cells (Fig. 5a). Additionally, on the 32nd day after starting steroid treatment, myocardial repeat biopsy revealed an admixture of CD4+ and CD8+ T cells and many histiocytes/macrophages within the myocardial inflammation suggested chronic smoldering myocarditis (Fig. 5b). Because of the persisting inflammation, the patient was placed under long-term prednisone therapy (15 mg/day). Cardiac MRI showed that the left ventricular wall was extensively fibrotic, and myocardial scintigraphy revealed that the ejection fraction had dropped to 20% (Fig. 6). The tumor reduction tendency of immunotherapy continued in both the remaining primary lesion and multiple lung metastases (Fig. 7). Since the patient’s performance status remained limited due to the chronic heart failure and many of these tumor responses were sustained over months, we decided to observe and not to reintroduce immunotherapy. Figure 8 shows a summary of his clinical course.

## Discussion and conclusions

We presented a case of advanced renal cell carcinoma in which drug-induced myocarditis rapidly worsened after immunotherapy. Immunotherapy with immune checkpoint inhibitors (ICIs) represents a shifting paradigm in the management of patients with advanced malignancies. As the clinical use of ICI therapy rapidly increases globally, the management of irAEs is becoming important. The growing clinical use of ICIs for different cancers has been accompanied by case reports and series of patients experiencing severe cardiac side effects. ICI-associated cardiac adverse reactions are rare but may present as severe myocarditis, with mortality rates ranging from 22.5% to 39.7% [2, 3].

In a pharmacovigilance study that ended in April 2016, myocarditis was noted in 0.09% of patients on a single ICI and in 0.27% of patients receiving combination therapy [4]. Additionally, in a recent pharmacovigilance study that ended in January 2018, 0.41% of patients that were prescribed anti-PD-1 or anti-PD-L1 monotherapy developed myocarditis compared with those prescribed anti-CTLA-4 monotherapies (0.07%) and combination ICIs (1.33%) [5]. In contrast, in a small registry of ICI-related myocarditis, the prevalence was lowest with anti-PD-1 agent (0.5%); however, it was higher with anti-PD-L1 (2.4%) and anti-CTLA-4 monotherapy (3.3%). Moreover, the prevalence of myocarditis was 2.4% with combined anti-PD-1 and anti-CTLA-4 therapy compared with 1.0% with combined anti-PD-L1 and anti-CTLA-4 therapy [6]. The true incidence of ICI-associated myocarditis is possibly underestimated given the lack of routine cardiac monitoring during treatment in most immunotherapy trials.

Many patients have no symptoms, while others may develop nonspecific symptoms such as fatigue and chest pain, or present with acute heart failure or sudden death. The mechanisms underlying the occurrence of irAEs with ICI therapy have not been fully elucidated. One possible pathophysiologic mechanism is that cardiac myocytes may share targeted antigens with the tumor, consequently becoming the targets of activated T cells, and resulting in lymphocytic infiltration with downstream heart failure and conduction abnormalities [4]. Nevertheless, the risk factors for ICI-associated myocarditis are not well understood [7].

The diagnosis of myocarditis can be challenging and requires a very high index of clinical suspicion. If a patient has symptoms suggestive of myocarditis, ECG and troponin level assessment should be immediately performed as initial diagnostic tests [8]. Additional testing, including coronary angiography and a viral serology panel, may be considered to exclude other causes. Endomyocardial biopsy is currently considered to be the standard diagnostic procedure for ICI-associated myocarditis. However, the test may not be performed as first line because of its invasive nature, risk of cardiac perforation, and localized nature of the biopsy sample, which could result in false-negative results [9]. A cardiac MRI can achieve a diagnosis in the early course of the disease when biopsy is not feasible, as it can show signs of myocardial inflammation [10].

High-dose glucocorticoids (methylprednisolone 1000 mg/day for the first 3 days followed by oral prednisone 1 mg/kg) are recommended as first-line therapy in the acute phase [9], but several reports have suggested that corticosteroids alone might not be sufficient to resolve the immune-mediated side effects [3]. For stable patients, immunosuppression therapy such as tacrolimus, mycophenolate mofetil, and infliximab may be considered, and anti-thymocyte globulin or intravenous immunoglobulin may be used in the management of toxicities for steroid-refractory or unstable patients [9].

Of 40 notable cases that developed myocarditis with ICI therapy for various malignancies, this case is the second report of myocarditis related to the treatment of urological cancer including renal cell carcinoma [3]. Therefore, this phenomenon may not be well known among urologists. Knowledge of this adverse event is crucial because of the notably high risk of mortality. Our experience highlights the importance of a high index of suspicion, prompt diagnosis, and early intervention for patients who present with cardiac abnormalities and possible myocarditis after receiving immunotherapy.

In conclusion, ICIs are approved for a wide range of malignancies. Cardiotoxicity is a rare event but is considered to be more frequent than previously known, and can become life-threatening, making it an important consideration for cardiologists, oncologists, and urologists. It is important for providers across specialties to be aware of both common and rare adverse effects associated with these agents.

## Data Availability

Not applicable.

## Abbreviations

irAEs: Immune-related adverse events
CT: Computed tomography
MRI: Magnetic resonance imaging
ECG: Electrocardiogram
ICIs: Immune checkpoint inhibitors
